# Diabetisches Fußsyndrom – Teil 1: Definition, Pathophysiologie, Diagnostik und Klassifikation

**DOI:** 10.1007/s00104-020-01301-9

**Published:** 2020-11-10

**Authors:** G. Rümenapf, S. Morbach, U. Rother, C. Uhl, H. Görtz, D. Böckler, C.‑A. Behrendt, D. Hochlenert, G. Engels, M. Sigl, Kommission PAVK und Diabetisches Fußsyndrom der DGG e. V.

**Affiliations:** 1 Oberrheinisches Gefäßzentrum Speyer, Klinik für Gefäßchirurgie, Diakonissen-Stiftungs-Krankenhaus, Paul-Egell-Straße 33, 67346 Speyer, Deutschland; 2 Abteilung Diabetologie und Angiologie, Fachbereich Innere Medizin, Marienkrankenhaus gGmbH Soest, Soest, Deutschland; 3 Gefäßchirurgische Abteilung, Universitätsklinikum Erlangen, Erlangen, Deutschland; 4 Klinik für Gefäßchirurgie und Endovaskuläre Chirurgie, Universitätsklinikum Heidelberg, Heidelberg, Deutschland; 5 Klinik für Gefäßchirurgie, Bonifatius Hospital Lingen, Lingen, Deutschland; 6 Klinik und Poliklinik für Gefäßmedizin, Universitätsklinikum Hamburg-Eppendorf, Hamburg, Deutschland; 7 Centrum für Diabetologie, Endoskopie und Wundheilung Köln, Köln, Deutschland; 8 Chirurgische Praxis am Bayenthalgürtel, Köln, Deutschland; 9 1. Medizinische Klinik, Abteilung für Angiologie, Universitätsklinik Mannheim, Mannheim, Deutschland

**Keywords:** Amputation, Diabetes mellitus, Diabetisches Fußsyndrom, Diabetische Polyneuropathie, Periphere arterielle Verschlusskrankheit, Amputation, Diabetes mellitus, Diabetic foot syndrome, Diabetic polyneuropathy, Peripheral arterial occlusive disease

## Abstract

In Deutschland leben ca. 8 Mio. Menschen mit Diabetes mellitus. Eine Spätfolge dieser Erkrankung ist das diabetische Fußsyndrom (DFS), dessen Prävalenz stark ansteigt. Es umfasst alle Veränderungen am Fuß als Folge der diabetischen Polyneuropathie sowie mikro- und makroangiopathischer (periphere arterielle Verschlusskrankheit, PAVK) Veränderungen. Jährlich entstehen ca. 250.000 neue diabetische Fußulzera. Diese werden oft zu chronischen Wunden. Trotz intensiver Bemühungen um Prävention, frühzeitige Diagnostik und stadiengerechte Wundbehandlung werden in Deutschland jährlich ca. 13.000 Majoramputationen bei Diabetikern durchgeführt. Bei konsequenter Therapie des DFS in interdisziplinären Zentren mit Ausschöpfung aller Möglichkeiten der Wundbehandlung, der Druckentlastung sowie einer arteriellen Revaskularisation kann die Majoramputationsrate um bis zu 80 % gesenkt werden. Durch eine geeignete Präventionsstrategie wäre die große Gefahr der Rezidivulzera geringer.

## Abstract (en)

There are ca. 8 million persons with diabetes mellitus living in Germany. A late sequelae of diabetes is the diabetic foot syndrome (DFS), the prevalence of which is greatly increasing. It comprises all alterations of the foot as a result of diabetic polyneuropathy as well as microvascular and macrovascular (peripheral arterial occlusive disease, PAOD) alterations. Many of the ca. 250,000 newly diagnosed diabetic foot ulcers per year become chronic wounds. Despite intensive efforts for prevention, early diagnosis and adequate wound care, ca. 13,000 persons with diabetes undergo major limb amputation in Germany every year. With consistent treatment in interdisciplinary centers and by exhausting all possible methods of wound treatment, pressure relief as well as arterial revascularization, the major amputation rate in patients with diabetic foot problems can be reduced by 80%. With a suitable strategy of prevention, the recurrence rate of foot ulcers would be reduced.

## Lernziele

Nach der Lektüre dieses Beitrags …

- kennen Sie Häufigkeit und Gefahren des diabetischen Fußsyndroms (DFS),
- verstehen Sie die Pathophysiologie des DFS,
- können Sie einen neuroischämischen diabetischen Fuß erkennen,
- können Sie für eine entsprechende Gefäßdiagnostik sorgen,
- kennen Sie die aktuellen Klassifikationen des DFS.

## Hintergrund

In Deutschland leben schätzungsweise 8 Mio. Menschen mit **Diabetes mellitus**

Diabetes mellitus

(DM) [1]. Mit zunehmender Diabetesdauer nehmen Folgeerkrankungen zu, die größtenteils auf mikro- und makrovaskulären Veränderungen beruhen. Eine davon ist das diabetische Fußsyndrom (DFS), ein Sammelbegriff für pathologische Veränderungen am Fuß von Menschen mit Diabetes mellitus.

Das DFS ist eine häufige, komplexe, kostenintensive und mitunter **lebensgefährliche Komplikation**

lebensgefährliche Komplikation

des DM [2, 3, 4]. Ulzera oder Nekrosen entstehen durch lokale Drucküberlastung bei eingeschränkter Schmerzempfindung als Folge der diabetischen Polyneuropathie (PNP). Häufig bestehen gleichzeitig Fuß- und Zehendeformitäten. In mehr als 50 % der Fälle kommt eine relevante periphere arterielle Verschlusskrankheit (PAVK) der Becken- und Beinschlagadern im Sinne einer kritischen Extremitätenischämie („critical limb ischemia“, CLI) hinzu, was die Wundheilung behindert und die hohe Zahl von **Majoramputationen**

Majoramputationen

verursacht [5].

Leitliniengerechte **interdisziplinäre Konzepte**

interdisziplinäre Konzepte

([6, 7, 8]; „time is tissue“) zur Prävention, frühzeitigen Diagnostik und **rechtzeitigen Revaskularisation**

rechtzeitigen Revaskularisation

haben dazu geführt, dass die Zahl von Majoramputationen kontinuierlich absinkt [9, 10]. Noch mehr Amputationen wären möglicherweise vermeidbar, wenn die Hauptgefahr für die Entstehung diabetischer Fußläsionen, nämlich die **fehlende Nozizeption**

fehlende Nozizeption

der Füße, frühzeitiger als bisher erkannt würde [11]. Der Zeitraum vom Auftreten bis zur Behandlung einer diabetischen Fußläsion durch einen gefäßmedizinischen Spezialisten muss nach Auffassung der Autoren in Deutschland [5] kürzer werden. Weiterhin muss noch konsequenter an eine Revaskularisation [12] und eine sinnvolle Rezidivprophylaxe gedacht werden.

In der vorliegenden Arbeit werden Epidemiologie, Pathophysiologie, Diagnostik und Klassifikation des DFS aus gefäßmedizinischer Sicht beschrieben. Besonderer Wert wird daraufgelegt, dass die Kernprobleme des DFS, also Neuropathie, PAVK und Fußdeformitäten, als Bedingungen und Auslöser des DFS rasch erkannt und leitliniengerecht untersucht werden.

## Definition

Das diabetische Fußsyndrom (DFS) ist eine lebenslange, gefährliche Komplikation des Diabetes mellitus, welche die Mobilität der Betroffenen bedroht und bei verzögerter oder ineffektiver Behandlung zum Verlust der Extremität führen kann. Da es sich um eine komplexe **chronische Erkrankung**

chronische Erkrankung

mit hoher Rezidivgefahr [2, 3] handelt, sollte beim DFS von „aktiven und inaktiven Phasen (‚Remission‘)“ anstatt von „Heilung“ gesprochen werden.

International wird das DFS als „Infektion, Ulzeration oder Gewebezerstörung am Fuß eines Menschen mit Diabetes mellitus in Verbindung mit neurologischen Störungen und/oder einer PAVK“ definiert [8]. In Deutschland gehören auch Vorboten eines Ulkus wie z. B. **Hornhautschwielen**

Hornhautschwielen

oder die trockene, **schuppige Haut**

schuppige Haut

dazu [7].

## Epidemiologie

Atherogene Risikofaktoren führen immer häufiger zu altersabhängigen Folgeerkrankungen, wie der **PAVK**

PAVK

[13]. Diese betrifft bevorzugt Menschen mit Diabetes mellitus, deren Zahl weltweit auf 500 Mio. geschätzt wird [14]. Mit zunehmender Diabetesdauer nehmen auch mikroangiopathische Folgeerkrankungen, wie die **diabetische PNP**

diabetische PNP

der Füße, an Häufigkeit zu. Dementsprechend gibt es immer mehr Menschen mit DFS, welches nach venösen Ulzera die zweithäufigste Ursache für chronische Wunden am Bein ist.

Jeder vierte Mensch mit Diabetes bekommt im Laufe seines Lebens ein DFS, bei ca. 3 % bestehen Ulzera im Fußbereich [15], die jährliche Inzidenz beträgt 2–6 % [3]. In Deutschland entstehen jährlich ca. 250.000 solcher oftmals chronischen Fußläsionen. Das DFS ist zudem die häufigste Ursache für **Ober- oder Unterschenkelamputationen**

Ober- oder Unterschenkelamputationen,

von denen in Deutschland jährlich über 13.000 bei dieser Patientengruppe durchgeführt werden [10]. Bei Menschen mit Diabetes kommt es über 40-mal häufiger zu Amputationen als bei Personen ohne Diabetes [16]. Nach 5 Jahren sind bis zu 80 % aller Menschen mit Diabetes nach einer Beinamputation verstorben, 3‑mal mehr als ohne Amputation [17] und wesentlich mehr als bei einem Karzinom [4].

Das DFS verursacht erhebliche Behandlungskosten [3, 4], **zeitintensive Krankenhausbehandlungen**

zeitintensive Krankenhausbehandlungen

[18] und für Gefäßmediziner ein zunehmendes Arbeitsvolumen. In den USA werden jährlich fast 240 Mrd. US-$ für die Diabetesbehandlung ausgegeben, davon ein Drittel für das DFS [4]. Damit ist die Behandlung des DFS in den USA so teuer wie die von Karzinomen, wobei deren 5‑Jahres-Mortalität mit 31 % geringer ist als die des DFS mit Minor- oder Majoramputation (56 %).

### Merke

- Das DFS ist die häufigste Ursache für Amputationen.
- Das DFS verursacht enorme Behandlungskosten.

## Suche nach Risikogruppen

Diabetologische Behandlungsstrategien konnten den progressiven Verlauf der Erkrankung und chronische diabetische Komplikationen bisher nicht erfolgreich aufhalten. Es wäre deshalb sinnvoll, zur Verhütung der klassischen Spätfolgen (z. B. Niereninsuffizienz, DFS) frühzeitig das Risikopotenzial des Patienten zu erkennen. So gibt es **Typ-2-Diabetes-Untergruppen**

Typ-2-Diabetes-Untergruppen

[19], die schon bei der Erstdiagnose ein erhöhtes Risiko für Nieren- oder Augenkomplikationen haben. Ob das auch für diabetische Neuropathie und PAVK gilt, ist bislang unbekannt.

## Pathogenese und Pathophysiologie

### Einteilung

Das DFS beruht auf neuropathischen, mikro- und makrovaskulären Störungen. Diese sind oft begleitet von Ödemen und septischen Thrombosen bei lokalen Infektionen und gestörter Immunabwehr.

**Figure 1:**
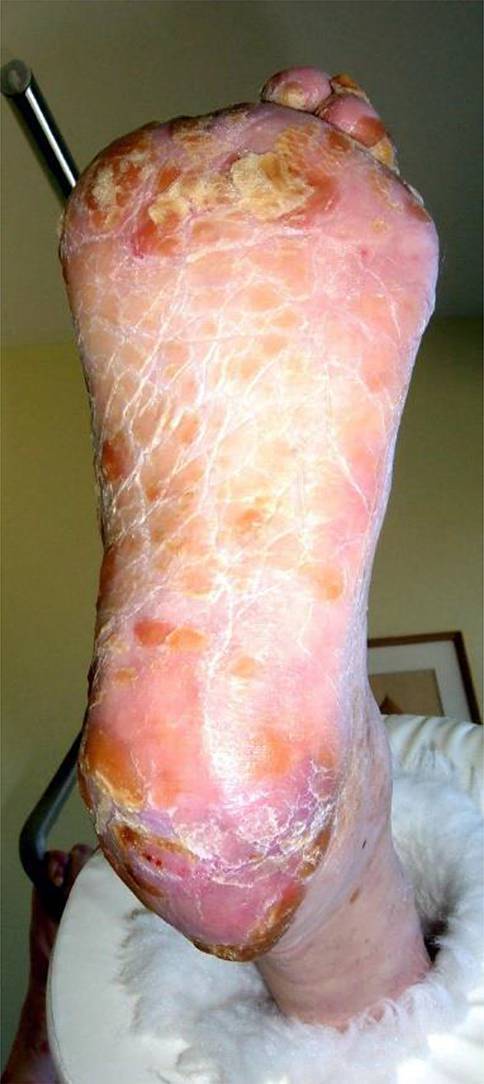

**Figure 2:**
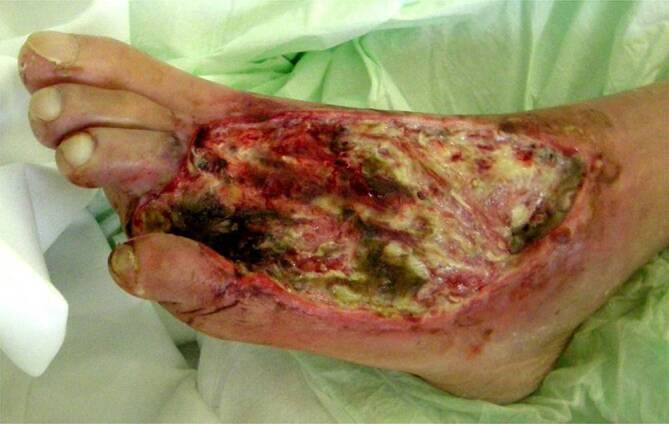

Läsionen am Fuß eines Menschen mit Diabetes werden nach der führenden Grunderkrankung unterteilt in:

- neuropathisch (Abb. 1),
- neuroischämisch (Abb. 2) und
- vorwiegend ischämisch.

Hinzu kommt die diabetische neuropathische Osteoarthropathie (**„Charcot-Fuß“**

**Figure 3:**
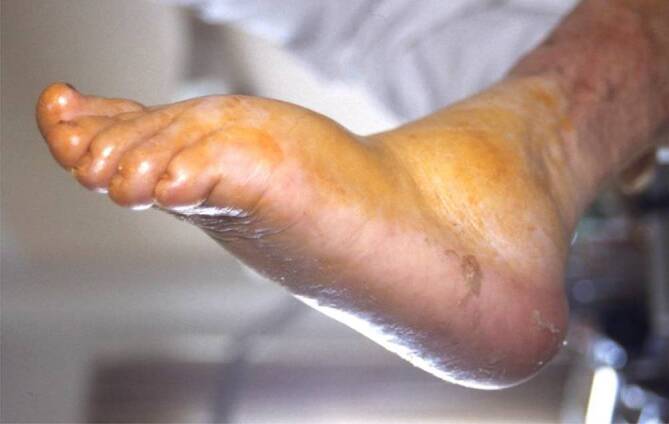

„Charcot-Fuß“,

DNOAP; Abb. 3*),* die mit oder ohne Ulzera auftreten kann.

### Risikofaktoren für das DFS

Der aufrechte Gang des Menschen führt zu einer hohen Druckbelastung der Füße. Schmerzen, gute Durchblutung und eine ungestörte Wundheilung schützen vor Verletzungen.

Beim Menschen mit DFS führen intrinsische und extrinsische Risikofaktoren dazu, dass diese **Schutzmechanismen fehlen**

Schutzmechanismen fehlen.

Nicht die Wunde oder der Charcot-Fuß sind chronisch, sondern die resistenzmindernden Bedingungen und lokalisationsbestimmenden Anlässe [20]. Deshalb müssen bei jeder diabetischen Fußläsion folgende Fragen gestellt werden:

„Warum besteht das Ulkus überhaupt?“ (**resistenzmindernde Bedingungen**

resistenzmindernde Bedingungen):

1. diabetische Neuropathie,
2. Mikro- und Makroangiopathie (z. B. PAVK),
3. Ödeme der Weichteile,
4. Atrophie des plantaren Fettpolsters,
5. Hornhautschwielen, Kallus,
6. Abwehrschwäche gegenüber Infektionen,
7. Depression,
8. Nichtbeachtung (Neglekt) bei „Leibesinselschwund“. Der betroffene Fuß wird neuropathiebedingt nicht mehr zum eigenen Körper gezählt, sondern als Teil der Umgebung und als „Problem des Arztes“ wahrgenommen.

**Figure 4:**
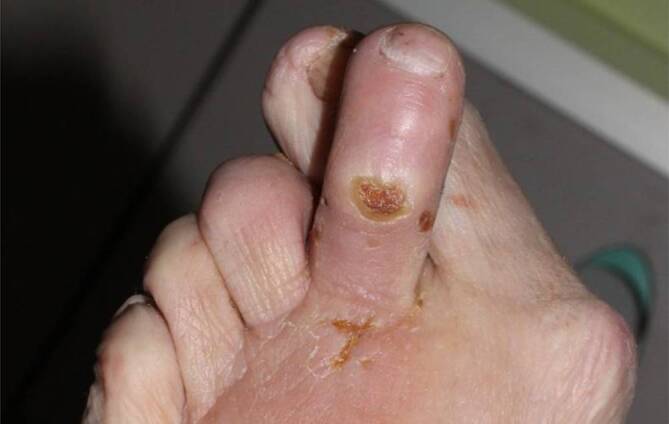

„Warum ist das Ulkus gerade an dieser Stelle?“ (**lokalisationsbestimmende Anlässe**

lokalisationsbestimmende Anlässe):

1. unpassende Schuhe,
2. Gegenstände im Schuh (z. B. Münzen),
3. Fuß- und Zehendeformitäten (Abb. 4), eingeschränkte Gelenkmobilität,
4. Verkürzung der Wadenmuskulatur mit Spitz- und Ballenfuß,
5. Adipositas,
6. Barfußgehen,
7. unsachgemäße Fußpflege,
8. Verbrennungen (Heizkissen bei „kalten Füßen“),
9. Stürze, Unfälle,
10. Spontanfrakturen bei diabetischer Ostoeoarthropathie (Charcot-Fuß).

#### Cave

- Häufig besteht beim Charcot-Fuß eine schwere PAVK.
- Der Charcot-Fuß wird meist zu spät erkannt.

„Warum heilt das Ulkus nicht?“:

1. fehlende Druckentlastung der Läsionen,
2. zu hohes Aktivitätsniveau,
3. unzureichende Nachsorge (Fußambulanz, Schuhversorgung, Podologie),
4. Nichtbeachtung der PAVK mit CLI,
5. unzureichend therapierte bakterielle Infektionen,
6. geringe Therapieadhärenz des Patienten.

Würden alle Regeln von** Wundbehandlung**

Wundbehandlung,

Revaskularisation und konsequenter **Druckentlastung**

Druckentlastung

beachtet, wäre das Behandlungsergebnis bei Menschen mit und ohne Diabetes womöglich vergleichbar. Verlauf und Behandlungsrealität sind beim DFS aber ernüchternd [3].

#### Merke

Beim DFS stellen sich 3 Fragen:

- Warum besteht es überhaupt?
- Warum an dieser Stelle?
- Warum heilt es nicht?

## Pathophysiologie und Symptomatik

### Schrittmacher: diabetische Polyneuropathie

Die wichtigste Ursache für die Entstehung von Fußulzera ist die diabetische PNP in Kombination mit **abnormer Druckbelastung**

abnormer Druckbelastung

(Abb. 1). Nahezu alle DFS-Patienten mit Fußulzera haben eine reduzierte oder fehlende Schmerzempfindung (Nozizeption, [11]). Kann ein Patient auf einem Fußulkus schmerzlos gehen, muss eine Neuropathie vorliegen [11, 20].

Die symmetrisch distal verteilte **sensorische Polyneuropathie**

sensorische Polyneuropathie

führt zu einer Verminderung des Empfindens für Vibration, Berührung, Druck, Schmerz und Temperatur (sog. Minussymptome). Druckstellen und Verletzungen werden vom Patienten nicht gespürt. Oft kommt es auch zu sog. Plussymptomen, wie Parästhesien („kalte Füße“) oder Schmerzen („painful painless leg“; [21]).

Die **motorische Neuropathie**

motorische Neuropathie

führt zur Atrophie der Fußbinnenmuskeln und zu einem Ungleichgewicht der Unterschenkelmuskulatur. Durch Überwiegen der Wadenmuskeln entstehen **Ballen- und Spitzfuß**

Ballen- und Spitzfuß

[3]. Die Zehenheber versuchen, den Kraftverlust der Fußhebermuskeln auszugleichen. Es entstehen Hammer- und Krallenzehen. Alle Deformitäten prädestinieren zu Druckulzera. Die Atrophie des Fußsohlenfetts verstärkt die Druckbelastung der Mittelfußköpfchen.

Die **autonome Neuropathie**

autonome Neuropathie

(„Autosympathektomie“) führt zu **verminderter Schweißsekretion**

verminderter Schweißsekretion,

trockener, rissiger Haut mit Hyperkeratosen, übermäßiger Schwielenbildung und zu trophischen Veränderungen der Zehennägel (Abb. 1 und 5). Die Haut ist durch die Öffnung von AV(arteriovenösen)-Shunts warm und täuscht eine gute Durchblutung des Fußes vor (s. unten).

**Figure 5:**
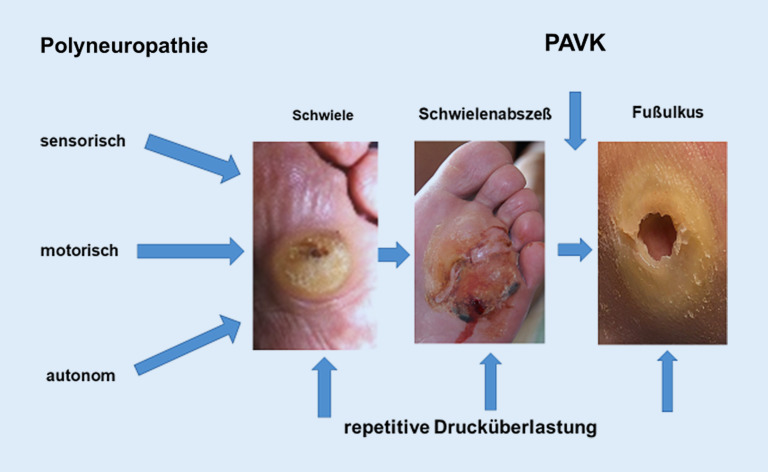

**Repetitive Drucküberlastung**

Repetitive Drucküberlastung

führt zu schmerzlosen Schwielen (Kallus) und subkallösen Blutungen (Abb. 5). Bei Infektion entstehen **Schwielenabszesse**

**Figure 6:**
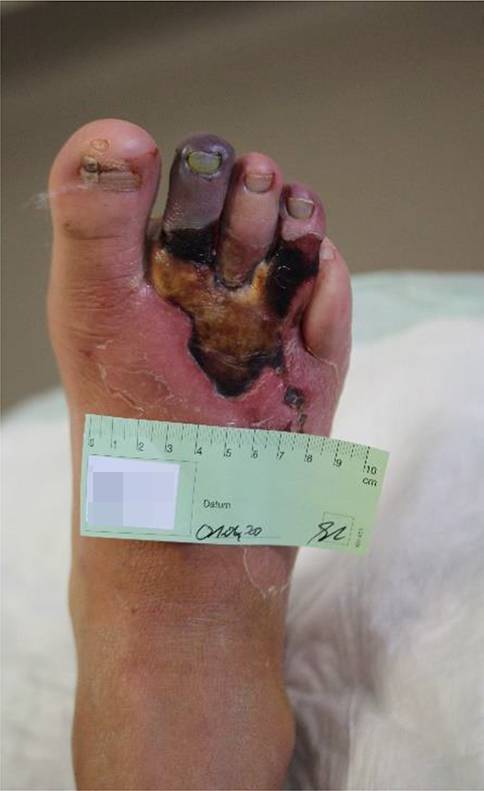

Schwielenabszesse,

die nach außen aufbrechen („Malum perforans“) oder tiefer gelegene Strukturen wie Knochen und Gelenke erfassen können (Abb. 6). Wenn eine relevante PAVK hinzukommt, stagniert die Wundheilung trotz konsequenter Druckentlastung und die Gefahr einer Gangrän oder Amputation steigt. Die PAVK wird jetzt behandlungspflichtig, obwohl die PNP die Fußläsionen verursacht hat.

#### Merke

- Die Hauptursachen von Fußulzera beim DFS sind Polyneuropathie und abnorme Druckbelastung.
- Die PAVK ist selten die Ursache, erschwert aber die Wundheilung.

Eine weitere Folge der Neuropathie ist die **diabetische Neuroosteoarthropathie**

diabetische Neuroosteoarthropathie

(DNOAP; [22]). Als Folge einer mechanischen Überlastung durch redundante Belastung bei fehlendem Schmerz kommt es zur **Demineralisierung**

Demineralisierung

und zu Spontanfrakturen des Fußskeletts. Auch eine abakterielle entzündliche Komponente wird diskutiert, des Weiteren eine trophische Störung des Knochens aufgrund der Denervierung.

**Figure 7:**
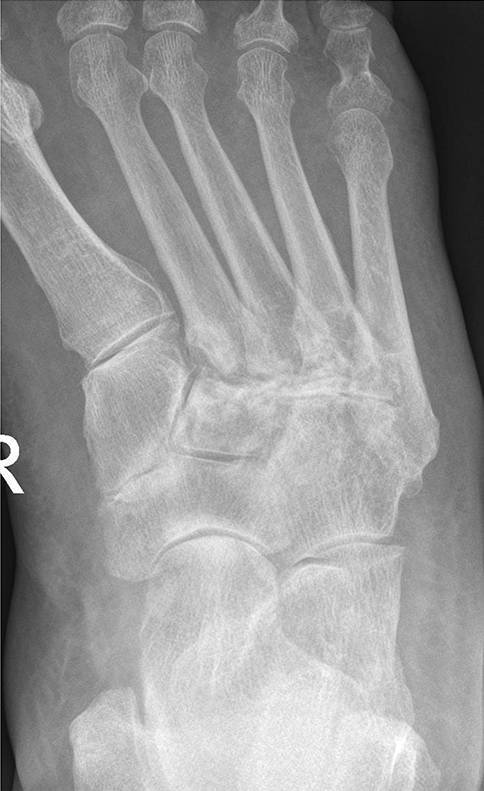

Es werden vier klinische Verlaufsstadien (Tab. 1) und fünf röntgenologische Befallsmuster (Tab. 2; Abb. 7) unterschieden, wobei es Mischbilder gibt.

**Table 1:** 

Verlaufsstadium	Klinik und Befunde
I	*Akutes Stadium:* Rötung, Schwellung, Überwärmung des Fußes
II	*Desintegration:* Knochen- und Gelenkveränderungen, Frakturen
III	*Knöcherne Konsolidierung* mit Fußdeformität: z. B. Plattfuß, Wiegefuß (Tintenlöscher)
IV	Zusätzliche* plantare Fußläsion*

**Table 2:** 

Sanders	Befall
1	Zehen bis Metatarsalia
2	Lisfranc-Gelenk
3	Chopart-Gelenk
4	Sprunggelenke
5	Kalkaneus

In der Gefäßchirurgie werden häufig Patienten mit DNOAP behandelt, bei denen neben der diabetischen PNP gleichzeitig eine schwere PAVK besteht. Die DNOAP wird oftmals erst erkannt, wenn das Endstadium mit Zusammenbruch des Fußskeletts zum **Plattfuß**

Plattfuß

erreicht ist.

#### Merke

- Das DFS wird in neuropathisch, neuroischämisch und ischämisch eingeteilt.
- Die meisten Ulzera sind neuroischämisch.

### Durchblutungsstörungen beim DFS

Die PAVK ist beim DFS die Hauptursache für das Nichtabheilen der Wunden und für die Majoramputation.

Mehr als 50 % aller Patienten mit DFS haben eine relevante PAVK. Im Gegensatz zu Menschen ohne Diabetes sind in 70 % der Fälle vorwiegend die **Unterschenkelarterien**

**Figure 8:**
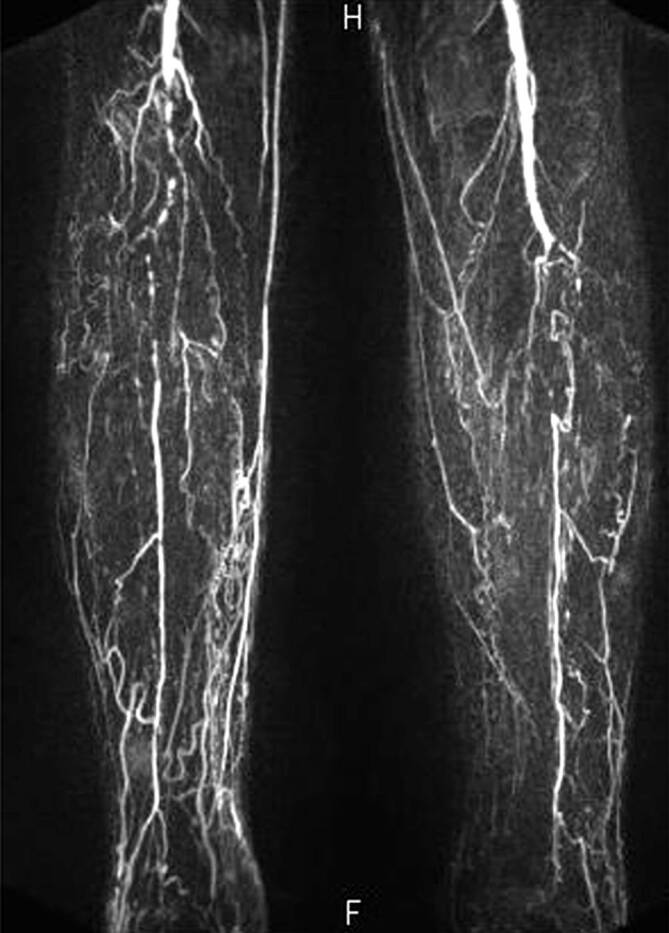

Unterschenkelarterien

betroffen (Abb. 8). Die Fußarterien sind oft noch erhalten [23], was die Möglichkeit **pedaler Bypässe**

pedaler Bypässe

eröffnet. Verschlussprozesse mehrerer Gefäßetagen sind allerdings nicht selten. Die A. profunda femoris ist häufig massiv langstreckig verändert, die femoropoplitealen Arterien aber oftmals nur wenig betroffen [24], sodass der Kniekehlenpuls bei vielen Patienten mit DFS noch tastbar ist.

Aufgrund der sensiblen Neuropathie bleibt die CLI häufig unerkannt. Sie manifestiert sich erst durch **Fußläsionen**

Fußläsionen

mit rasch fortschreitenden **Infektionen**

Infektionen.

Die Stadieneinteilungen der PAVK (z. B. nach Fontaine oder Rutherford) orientieren sich an Schmerzen und Gewebeverlust und sind beim DFS deshalb unbrauchbar. Rein ischämische, schmerzhafte Ulzera sind bei Diabetikern selten.

**Figure 9:**
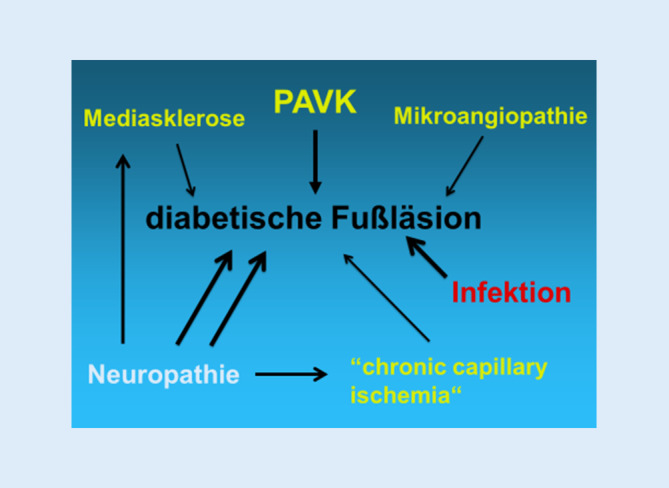

Zur PAVK gesellen sich beim DFS weitere Faktoren, welche die Ischämie des Fußgewebes verstärken, die Wundheilung stören und die PAVK wesentlich bedrohlicher machen als beim Nichtdiabetiker (Abb. 9). Schon beim rein neuropathischen DFS ist die **Sauerstoffversorgung**

Sauerstoffversorgung

des Gewebes schlechter als bei einem gesunden Fuß.

Die **diabetische Mikroangiopathie**

diabetische Mikroangiopathie

mit Verdickung der kapillären Basalmembran behindert die Sauerstoffdiffusion, erhöht aber nicht den Gefäßwiderstand und schließt eine Revaskularisation nicht aus. Sie ist ein unabhängiger Risikofaktor für eine Majoramputation [25].

Kälte führt normalerweise zu einer präkapillären Vasokonstriktion und Öffnung von arteriovenösen Shuntgefäßen der Haut. Bei diabetischer PNP tritt das infolge der autonomen Neuropathie auch ohne Abkühlung ein [26]. Der Fuß erscheint gut durchblutet, es besteht aber eine **chronische kapillare Ischämie**

chronische kapillare Ischämie.

Die Verkalkung der Tunica media (**Mediasklerose**

**Figure 10:**
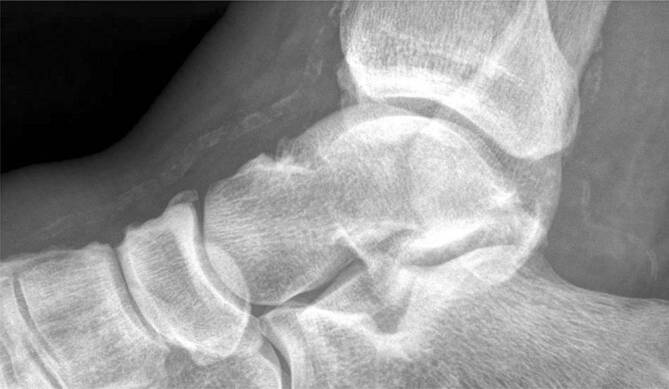

Mediasklerose,

Abb. 10) ist mit der autonomen PNP vergesellschaftet, vermindert die Elastizität der Gefäßwand, beschleunigt die PAVK, behindert die Sauerstoffversorgung des Gewebes und bedeutet ein erhöhtes Ulkus- und Amputationsrisiko [27]. Aufgrund der Inkompressibilität der Gefäßwand führt die Mediasklerose zu falsch-hohen Verschlussdrücken. Der Knöchel-Arm-Index („ankle-brachial index“, ABI) ist bei diesen Patienten nicht verwertbar. Eine schwere CLI wird dadurch oft übersehen.

#### Merke

- Mehr als 50 % aller Patienten mit DFS haben eine relevante PAVK.
- In 70 % sind die Unterschenkelarterien betroffen.

## Diagnostik

Details zur Anamneseerhebung und Fußuntersuchung (diabetische Grunderkrankung, Stoffwechsellage, Risikofaktoren für eine PAVK, Neuropathie, Gefäßstatus, Zustand des Fußskeletts) und zu Differenzialdiagnosen werden in den entsprechenden diabetologischen oder interdisziplinären Leitlinien beschrieben (z. B. [6, 7]). Der **Fuß-Dokumentationsbogen**

Fuß-Dokumentationsbogen

der Arbeitsgemeinschaft Fuß der Deutschen Diabetes Gesellschaft (DDG) ermöglicht die Beschreibung der Fußveränderungen [7].

### Anamnese.

Neben der Schilderung der aktuellen Fußprobleme sollten die Dauer und die derzeitige Behandlung des Diabetes mellitus erfragt werden. Ebenso sollte nach **Begleiterkrankungen**

Begleiterkrankungen

aus dem Umfeld der diabetischen Spätfolgen und des metabolischen Syndroms gefragt werden.

### Inspektion, Palpation.

Neben der Inspektion und Beschreibung der Läsion bezüglich Lokalisation, Größe, Tiefenausdehnung, Zeichen von Infektion und Ischämie (s. Klassifikation), möglichst mit gleichzeitiger Fotodokumentation, sollte auch auf Veränderungen der **Fuß- und Zehenform**

Fuß- und Zehenform

geachtet werden (z. B. Charcot-Fuß). Typische Lokalisationen ischämischer Läsionen sind Zehen, Fußaußenkante, Strecksehnen des Fußrückens, Ferse (bei bettlägerigen Patienten), Knöchel.

Qualität, Farbe und Temperatur der Haut werden im **Seitenvergleich**

Seitenvergleich

beurteilt. Ein warmer, rosiger Fuß schließt bei gleichzeitiger PNP eine PAVK nicht aus.

Die Fußläsion selbst muss ausgetastet werden (unterminierte Ränder, Knochenbeteiligung?). Fisteln werden sondiert („probe to bone“).

Auch sollten die **Schuhe**

Schuhe

des DFS-Patienten überprüft werden, da die meisten Fußläsionen durch unpassende Schuhe entstehen [11].

### Diabetische PNP

Bei Menschen mit DFS muss nach den folgenden Symptomen gefragt werden:

- Schwächegefühl (Ermüdung, Erschöpfung),
- Krämpfe,
- brennende oder stechende Schmerzen,
- Hyperästhesien,
- Parästhesien (Elektrisieren, „Ameisenlaufen“),
- Taubheitsgefühl,
- Temperaturmissempfindung.

Wichtig ist, ob die Symptome nur **tagsüber**

tagsüber

oder auch **nachts**

nachts

vorhanden sind und ob sie sich beim Gehen, Stehen, Sitzen oder Hinlegen bessern. Bei der Untersuchung der Füße muss auf klinische Zeichen der PNP wie Muskelatrophien, Hyperkeratosen etc. geachtet werden. Im Rahmen der Differenzialdiagnostik müssen andere Ursachen einer PNP erfragt werden (z. B. **Alkoholabusus**

Alkoholabusus).

Zusätzlich werden geprüft:

- Achillessehnenreflex,
- Vibrationsempfinden mit der Rydell-Seiffer-Stimmgabel (128 Hz),
- Berührungsempfindlichkeit mit dem 10-g-Monofilament (Semmes-Weinstein),
- Temperaturempfinden (z. B. Tiptherm),
- ggf. Pinprick-Test zur Messung der Nozizeption [10]*.*

Die Ergebnisse können in Form eines **Neuropathiesymptomscores**

Neuropathiesymptomscores

zusammengefasst werden [2]. Der **Pinprick-Test**

Pinprick-Test

bietet eine Möglichkeit, eine individuelle Präventionsstrategie gegen Fußläsionen zu entwickeln [11].

### Charcot-Fuß

Bei Verdacht auf eine DNOAP müssen **Nativröntgenaufnahmen**

Nativröntgenaufnahmen

des gesamten Fußes in zwei Ebenen unter **Vollbelastung**

Vollbelastung

gemacht werden, um eine Aussage über die funktionellen Auswirkungen der knöchernen Veränderungen zu treffen. Häufig sieht man Knocheneinbrüche und eine Entkalkung des Fußskeletts. Im frühen „Entzündungsstadium“ (Rötung, Schwellung und Überwärmung des Fußes), welches eine bakterielle Entzündung vortäuschen kann, sind auf Röntgenbildern noch keine Skelettveränderungen erkennbar. Findet sich eine Eintrittspforte für Bakterien, so ist eine **phlegmonöse Entzündung**

phlegmonöse Entzündung

wahrscheinlicher als die DNOAP. In diesem Fall sollte eine Magnetresonanztomographie durchgeführt werden. Sie kann typische Knochenödeme zeigen und die wichtigste Differenzialdiagnose, den abszedierenden infizierten diabetischen Fuß, davon abgrenzen. Auch bei Patienten mit DNOAP sollte die Durchblutung der Beine immer systematisch untersucht werden (s. unten).

### Gefäßdiagnostik beim DFS

Bei DFS muss immer an die PAVK gedacht werden, wenn eine Fußläsion trotz adäquater Wundbehandlung nicht heilt [6, 7, 8]. Deshalb ist bei allen Patienten mit DFS die Erhebung des **Gefäßstatus beider Beine**

**Figure 11:**
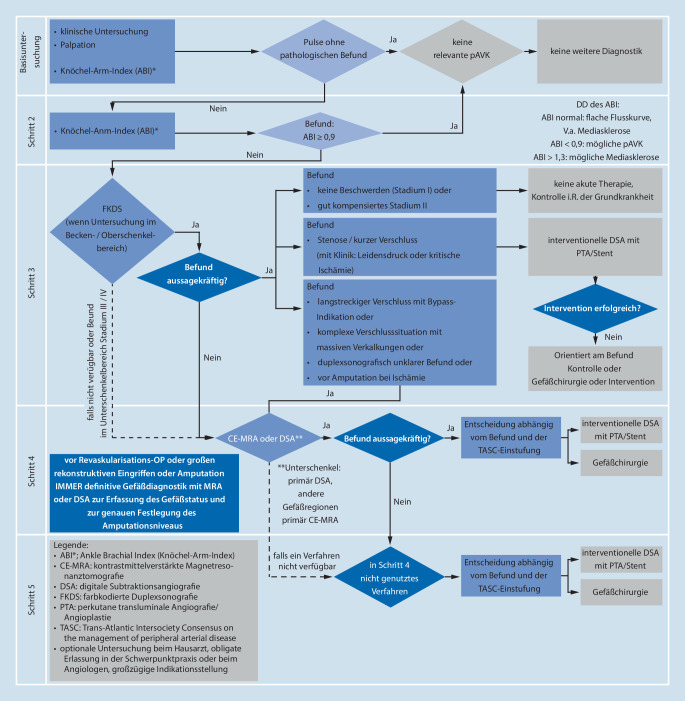

Gefäßstatus beider Beine

notwendig*.* Ein Stufenschema (Abb. 11) dient als Handlungsanleitung [6]. Zusätzlich sollte nach früheren Angiographien, Dilatationen und Gefäßoperationen gefragt werden. Entsprechende Narben an den Beinen weisen darauf hin.

Durch das **Tasten der Pulse**

Tasten der Pulse

am entkleideten Patienten in der Leiste, Kniekehle, am Innenknöchel und am Fußrücken kann die Etage des hauptsächlichen Gefäßproblems lokalisiert werden. Das Tasten der Knöchelpulse unter Zeitdruck ist wenig zuverlässig [28].

Es folgt die **dopplersonographische Messung**

dopplersonographische Messung

der Verschlussdrücke der Fußarterien (A. tibialis posterior, A. dorsalis pedis). Diese werden mit dem systolischen Blutdruck am Oberarm verrechnet und als **Knöchel-Arm-Index**

Knöchel-Arm-Index

(ABI) dokumentiert. Werte unter 0,9 lassen auf eine PAVK schließen. Sehr häufig (>50 %) ist der ABI beim DFS aufgrund einer Mediasklerose nicht verwertbar (ABI >1,3). Nur ein ABI <0,6 gilt als Hinweis für eine CLI [8]. Zuverlässiger ist die Zehendruckmessung. Um rasch eine CLI auszuschließen, bietet sich die **hydrostatische Zehendruckmessung**

hydrostatische Zehendruckmessung

(„pole pressure test“) an [29].

Aussagekräftiger als die obigen Methoden ist die **bidirektionale Continuous-wave(CW)-Dopplersonographie**

bidirektionale Continuous-wave(CW)-Dopplersonographie.

Kurvenform (mono-, bi-, triphasisch), Amplitude und Breite der Flusskurve und die Analyse des Frequenzspektrums weisen auf vorgeschaltete arterielle Verschlussprozesse hin. Triphasische CW-Strömungsgeschwindigkeitskurven sind ein zuverlässiger Hinweis, dass keine schwerwiegende Ischämie besteht.

Die wichtigste Untersuchung ist die **farbkodierte Duplexsonographie**

farbkodierte Duplexsonographie

(FKDS) der Beinarterien. Sie erlaubt neben der genauen morphologischen Beschreibung der Gefäßanatomie auch eine Quantifizierung des Stenosegrades. Geübte Untersucher können die Arterien des Beckens und Oberschenkels und, abhängig von der Gerätequalität und der Qualifikation und dem Zeitdruck des Untersuchers, die Unterschenkel- und Fußarterien in einem vernünftigen Zeitrahmen beurteilen. Die kruralen Arterien lassen sich aufgrund der häufig vorliegenden Mediaverkalkung mit FKDS nicht immer eindeutig beurteilen.

Die FKDS kann direkt zur antegraden digitalen Subtraktionsangiographie (DSA) in Interventionsbereitschaft überleiten.

Bleiben nach der FKDS noch Fragen zum Gefäßstatus, sollte eine Kontrastmittel(KM)-verstärkte Magnetresonanzangiographie (**„contrast-enhanced MRA“**

„contrast-enhanced MRA“,

ce-MRA) der Becken- und Beinarterien durchgeführt werden. Sie ermöglicht eine exakte Darstellung der Unterschenkel- und Fußarterien inkl. der zeitlichen Auflösung der Strömungsdynamik. Bei Patienten mit **Herzschrittmacher**

Herzschrittmacher

ist die ce-MRA meist unmöglich. Wegen toxischer Effekte mancher gadoliniumhaltiger Kontrastmittel bei **niereninsuffizienten Patienten**

niereninsuffizienten Patienten

mit der Gefahr der potenziell tödlichen nephrogenen systemischen Fibrose wird sie bei einer glomerulären Filtrationsrate (GFR) unter 60 ml/min × 1,73 m^2^ nur nach Einzelfallbewertung durchgeführt.

Obwohl die **digitale Subtraktionsangiographie**

digitale Subtraktionsangiographie

(DSA) der Becken- und Beinarterien am präzisesten ist, wird sie nur durchgeführt, wenn die bisher genannten Verfahren keine eindeutigen Ergebnisse erbracht haben oder nicht angewendet werden können. Die DSA birgt Gefahren durch die **Invasivität**

Invasivität

und die Gabe **jodhaltiger KM**

jodhaltiger KM

(Jodallergie, KM-induzierte Nephropathie, Hyperthyreose). Menschen mit Diabetes haben bei der Gabe von jodhaltigem Kontrastmittel auch bei normalen Kreatininwerten im Serum eine erhöhte Gefahr für eine KM-Nephropathie. Als Kontrastmittel kann bei fortgeschrittener Niereninsuffizienz **Kohlendioxid**

Kohlendioxid

verwendet werden. Am Unterschenkel stößt dieses Verfahren allerdings oft an seine Grenzen.

Die **Computertomographie(CT)-Angiographie**

Computertomographie(CT)-Angiographie

hat wie die DSA den Nachteil hoher jodhaltiger KM-Gaben. Oft ist die Beurteilbarkeit stark verkalkter Arterien für die Planung einer Revaskularisation nicht möglich. Beides spricht gegen ihren Einsatz bei Patienten mit DFS.

Eine chronisch venöse Insuffizienz (Varizen, postthrombotisches Syndrom) kann zu chronischen Ulzera führen oder (neuro-)ischämische Ulzera bei DFS verstärken (Ulcus cruris mixtum). Deshalb muss immer auch der **Zustand der Beinvenen**

Zustand der Beinvenen

mittels FKDS untersucht werden.

#### Merke

- Beim DFS muss nach Zeichen der Neuropathie und einer PAVK gesucht werden.
- Der Knöchel-Arm-Index (ABI) ist beim DFS unbrauchbar.
- Entscheidend für die weitere Diagnostik sind Duplexsonographie, MR-Angiographie, und die DSA in Interventionsbereitschaft.

#### Cave

- DFS-Patienten haben häufig Nierenfunktionsstörungen.
- Zur DSA sollte CO_2_ statt Jodhaltiger Kontrastmittel gegeben werden.

## Klassifikation des DFS

Diabetische Fußläsionen sollten nach den Kriterien Ausmaß, Lokalisation, Wundheilungsstadium, Infektion, Neuropathie und Ischämie dokumentiert und in einem Klassifikationssystem eingeordnet werden. Dies sollte der fachlichen Kommunikation dienen. Je einfacher und verständlicher, umso praxistauglicher ist das Klassifikationssystem. Komplizierte Klassifikationen werden wissenschaftlich genutzt (s. unten).

Die Einteilungen nach **Wagner und Armstrong**

**Figure 12:**
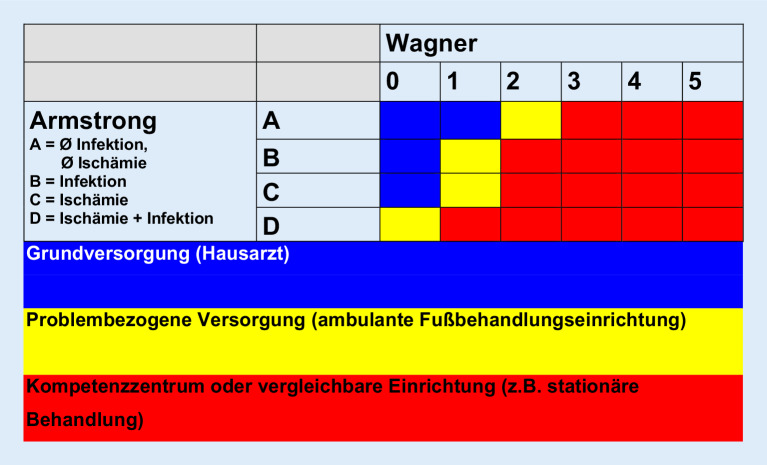

Wagner und Armstrong

(Tab. 3; Abb. 12) werden in der klinischen Praxis am häufigsten verwendet [6, 7]. Während sich erstere auf Ausdehnung, Lokalisation und Tiefe der Wunde beschränkt, berücksichtigt letztere zusätzlich die Pathogenese (Ischämie und Infektion). Die Deutsche Diabetes Gesellschaft (DDG) verwendet diese Klassifikation. Der Aufwand ist gering, es fehlen jedoch die Bewertung der Neuropathie und eine wissenschaftliche Evaluation.

**Table 3:** 

Grad	
0	Keine Läsion, ggf. Fußdeformation oder Zellulitis
1	Oberflächliche Ulzeration
2	Tiefes Ulkus bis zur Gelenkkapsel, zu Sehnen oder Knochen
3	Tiefes Ulkus mit Abszedierung, Osteomyelitis, Infektion der Gelenkkapsel
4	Begrenzte Nekrose im Vorfuß- und Fersenbereich
5	Nekrose des gesamten Fußes

Die relativ einfache **SINBAD-Klassifikation**

SINBAD-Klassifikation

[30] bewertet den Ort des Ulkus (S, „site“), Ischämie (I), Neuropathie (N), bakterielle Infektion (B) und Flächenausdehnung (A, „area“) sowie Tiefe (D, „depth“) des Ulkus.

Um das Risiko für eine Amputation und die Prognose mit einer CLI abschätzen zu können, wurde das **„WIFI-System“**

„WIFI-System“

**Figure 13:**
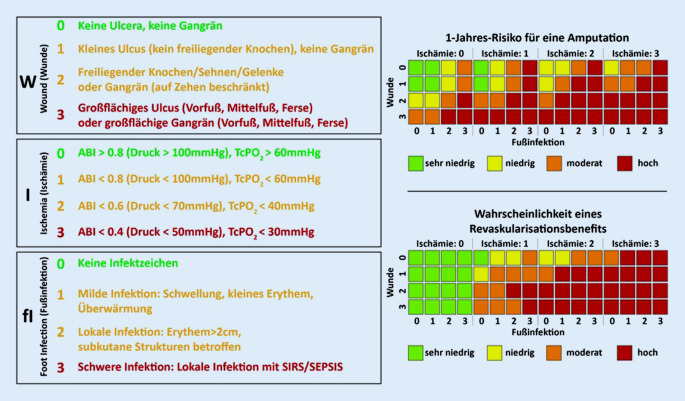

[31] entwickelt (Abb. 13), welches die Kriterien Wunde, Ischämie und Fußinfektion beinhaltet („wound“, „ischemia“, „foot infection“, WIFI). Es basiert auf der Klassifikation der Infectious Disease Society of America (IDSA)/International Working Group on the Diabetic Foot (IWGDF) zur Erfassung der Infektion. Amputationsraten und die Abheilungsdauer der Wunde korrelieren mit WIFI. Das WIFI-System wird vor allem wissenschaftlich genutzt. Es fehlt das Kriterium „Neuropathie“.

Die IWGDF empfiehlt SINBAD für den klinischen Gebrauch und für den Vergleich von Ergebnissen (Audit) und das WIFI-System zur Erfassung von Infektion und Perfusion und zur Abschätzung des Vorteils einer Revaskularisation [33]. Kein Klassifikationssystem kann die Prognose eines Ulkus vorhersagen [8].

### Merke

- Die Beschreibung diabetischer Fußläsionen sollte Ausmaß, Lokalisation, Wundheilungsstadium, Infektion, Neuropathie und Ischämie berücksichtigen.
- In Deutschland wird die Klassifikation nach Wagner/Armstrong empfohlen.

## Fazit für die Praxis

- Bei Menschen mit Diabetes und Fußproblemen muss frühzeitig an die Risikofaktoren Polyneuropathie (PNP) und periphere arterielle Verschlusskrankheit (PAVK) gedacht werden. Deshalb sind Kenntnisse der Pathophysiologie des diabetischen Fußsyndroms (DFS) wichtig.
- Die körperliche Untersuchung beinhaltet die Erhebung der Pulsstatus der Beine, die Suche nach einer PNP, die Untersuchung der Wunden und die Beurteilung des Fußes hinsichtlich begünstigender Deformitäten. Dabei müssen folgende Fragen gestellt werden: Warum besteht die Wunde? Warum gerade an dieser Stelle? Warum heilt sie nicht?
- Bei Verdacht auf eine begleitende PAVK muss sofort ein Gefäßspezialist eingebunden werden.
- Dann müssen alle Methoden der apparativen und bildgebenden Diagnostik ohne Zeitverzögerung genutzt werden. Hierzu zählen die farbkodierte Duplexsonographie und angiographische Methoden inkl. der CO_2_-Angiographie.
- Die dopplersonographische Bestimmung des Knöchel-Arm-Index (ABI) ist beim DFS in mindestens der Hälfte der Fälle unzuverlässig. Ein normaler ABI schließt beim DFS eine PAVK nicht aus.
- Fußläsionen beim DFS sollten gut dokumentiert und mit einem geeigneten System klassifiziert werden. In Deutschland wird meist die kombinierte Wagner-Armstrong-Klassifikation verwendet.

## CME-Fragebogen

Menschen mit diabetischem Fußsyndrom (DFS) kommen öfters in Ihre Praxis. Welche Antwort ist richtig?

Das DFS ist bei regelmäßiger Kontrolle ungefährlich und kann ohne spezielle fachärztliche Expertise behandelt werden.

Der Diabetes mellitus ist in Deutschland selten.

Menschen mit Diabetes werden ca. 40-mal häufiger amputiert als Menschen ohne Diabetes.

Die Gefahr für diese Patienten, die nächsten 5 Jahre nicht zu überleben, ist geringer als bei einem Karzinom.

Die Kosten für das Gesundheitssystem, die das DFS verursacht, sind geringfügig.

Ein Patient mit Diabetes mellitus und schmerzlosem Ulkus an der Fußsohle kommt in die chirurgische Sprechstunde. Wenn Sie die Gefahren des DFS kennen, wissen Sie, welche der folgenden Aussagen richtig ist:

Das Problem des diabetischen Fußsyndroms ist klinisch nicht relevant, es besteht für den Patienten wenig Gefahr.

Das diabetische Fußsyndrom zählt nicht zu den Spätfolgen des Diabetes mellitus.

Das Risiko einer Gangrän ist beim Diabetiker mindestens 20-mal höher als beim Nichtdiabetiker.

Die Amputationszahlen in Deutschland sind nicht besorgniserregend.

Durch Präventionsmaßnahmen lässt sich die Amputationsrate beim DFS nicht senken.

Ein Mensch mit Diabetes wird seit Monaten in einer chirurgischen Praxis behandelt. Welche Aussage ist richtig?

Ein Fußulkus bei Diabetikern ist selten für eine Klinikeinweisung verantwortlich.

Die Behandlung des diabetischen Fußsyndroms (DFS) verbraucht vergleichsweise wenig Ressourcen.

Das Schuhwerk spielt bei der Entstehung eines Fußulkus eine untergeordnete Rolle.

Die Inzidenz und die Prävalenz diabetischer Fußulzera liegen in Deutschland bei ca. 3 %.

Psychosoziale Faktoren spielen bei der Entwicklung des DFS keine Rolle.

Das diabetische Fußsyndrom wird im Hinblick auf die Ursache und auch auf die Therapie sinnvollerweise unterteilt in …

neuropathisch/nichtneuropathisch/unklar.

Typ-I-assoziiert/Typ-II-assoziiert.

neuropathisch/neuroischämisch/rein ischämisch.

psychogen/organisch/Mischtyp.

neuropathisch-osteoarthropathisch/neuropathisch ohne Knochenbeteiligung.

Welcher der Tests hat in der Neuropathiediagnostik *am wenigsten* Aussagekraft?

Prüfen des Achillessehnenreflexes

Warm-Kalt-Testung (Tiptherm)

Stimmgabeltest

Monofilament-Methode

Psychometrischer Test

Welche der genannten Ursachen ist für eine pathologisch hohe plantare Druckbelastung *am wenigsten* relevant?

Barfuß gehen

Ungeeignetes Schuhwerk

Ballenfußfehlstellung

Beinlängendifferenz (2 cm)

Fremdkörper im Schuh

Ein Patient mit diabetischem Fußsyndrom (DFS) und einem großen Ulkus an der Ferse kommt in die chirurgische Ambulanz und soll von Ihnen untersucht werden. Was müssen Sie beachten?

Bei jedem Patienten mit DFS sollte die arterielle Durchblutung der Beine überprüft werden.

Pulse tasten ist nicht routinemäßig erforderlich.

Eine digitale Subtraktionsangiographie (DSA) sollte bei jedem Diabetiker mit DFS zum Einsatz kommen, bei dem Verdacht auf eine Durchblutungsstörung der Beine besteht.

Die kontrastmittelverstärkte Magnetresonanzangiographie („contrast-enhanced MRA“, ce-MRA) ist die Methode der Wahl bei Patienten mit Niereninsuffizienz, insbesondere bei Dialysepflichtigkeit.

Die CO_2_-Angiographie hat aufgrund der eingeschränkten Darstellung der Arterien keine Bedeutung.

Ein Patient mit Diabetes mellitus und einer chronischen Wunde an der Ferse wird von Ihnen untersucht. Die Fußläsion sollte klassifiziert werden. Wie denken Sie darüber, was ist richtig?

Die Verwendung der Klassifikation ist zeitaufwendig und im klinischen Alltag nicht realisierbar.

Die Klassifikation von Wagner beinhaltet außer der Tiefenausdehnung auch die Pathogenese der Läsion (Ischämie, Infektion).

Die Wagner-Armstrong-Klassifikation erlaubt auch Aussagen über das Ausmaß der diabetischen Neuropathie.

Die Wagner-Armstrong-Klassifikation wird von der Deutschen Diabetes Gesellschaft empfohlen und ist Bestandteil ihres Fuß-Dokumentationsbogens.

Die WIFI(„wound“, „ischemia“, „foot infection“)-Klassifikation ist einfach und schnell anwendbar und sollte die Wagner-Armstrong-Klassifikation im täglichen Routinebetrieb ersetzen.

Sie vermuten bei einem Patienten mit diabetischem Fußsyndrom (DFS) eine Durchblutungsstörung. Welche Aussage ist richtig?

Mit der Farbduplexsonographie lassen sich die Becken- und Beingefäße gut untersuchen, sodass oft auf eine Becken-Bein-Übersichtsangiographie verzichtet werden kann.

Die Indikation zur digitalen Subtraktionsangiographie des betroffenen Beines in PTA(perkutane transluminale Angioplastie)-Bereitschaft setzt eine kontrastmittelverstärkte Magnetresonanz-Übersichtsangiographie voraus.

Der ABI („ankle-brachial index“) beträgt 1,4, sodass eine Durchblutungsstörung ausgeschlossen werden kann.

Eine Computertomographie-Angiographie ist schnell und bedenkenlos durchführbar und liefert zuverlässige Befunde.

Die Gefahr einer Nephropathie nach Gabe jodhaltiger Kontrastmittel ist bei Menschen mit DFS vernachlässigbar gering.

Eine motorische Neuropathie erkennt man bei einem Patienten mit diabetischem Fußsyndrom (DFS) an …

Krallenzehen.

Hallux valgus.

Hackenfuß.

Hypertrophie der Fußhebermuskulatur.

kräftigen Fußbinnenmuskeln.
